# CHARACTERIZING THE EFFECTS OF HOSTILE ARCHITECTURE ON THE HEALTH GOALS OF HOUSELESS ELDERS

**DOI:** 10.1093/geroni/igz038.2077

**Published:** 2019-11-08

**Authors:** Ian M Johnson

**Affiliations:** University of Washington, Seattle, Washington, United States

## Abstract

Over 12,000 residents of Seattle experienced houselessness in 2018—among them, 70% reported having health conditions, 17.5% were over 50, and over half do not access emergency housing services. Local governments increasingly use strategies aimed at deterring unhoused populations from using public space. This research aimed to characterize the effects of urban planning interventions on the health goals among older disabled adults experiencing houselessness. Agency-based focus groups were conducted with adults over 50 who self-identified as disabled and met the federal criteria for homelessness. Through participatory mapping methods, constituents identified places where opportunities and barriers toward achieving health goals were experienced. Findings indicate lived experiences of confinement, exclusion, and loss of autonomy as well as creative negotiation and reclamation of space. This research equips advocates and providers with spatial data to increase public awareness, enrich local advocacy efforts, and offer new methodologies for enhancing social work perspectives on place and aging.

